# Risk Factors for Delayed Inpatient Functional Recovery after Total Knee Arthroplasty

**DOI:** 10.1155/2015/167643

**Published:** 2015-04-16

**Authors:** Thomas J. Hoogeboom, Nico L. U. van Meeteren, Kristin Schank, Raymond H. Kim, Todd Miner, Jennifer E. Stevens-Lapsley

**Affiliations:** ^1^Department of Epidemiology and Centre for Care Technology Research (CCTR), Maastricht University, Maastricht, Netherlands; ^2^Healthy for Life, TNO, Leiden, Netherlands; ^3^Department of Physical Therapy, Porter Adventist Hospital, Denver, CO, USA; ^4^Department of Orthopedics, Porter Adventist Hospital, Denver, CO, USA; ^5^Department of Mechanical & Materials Engineering, University of Denver, Denver, CO, USA; ^6^Department of Physical Medicine and Rehabilitation, University of Colorado, Aurora, CO, USA

## Abstract

*Purpose*. To determine the predictive value of surgery-related variables for delayed inpatient functional recovery (i.e., ≥3 days to reach functional independence) after TKA. *Method*. 193 consecutive people undergoing TKA were included in this prospective cohort study. Inpatient functional recovery was measured daily using the Iowa Level of Assistance scale (ILAS). Two persons reviewed medical records to extract patient characteristics (i.e., age, sex, and BMI) and surgical factors (i.e., blood loss, tourniquet time, postoperative morphine use, and surgical experience). Odds ratios (OR) and area under the curves (AUC) were calculated to determine the predictive value of the putative factors and of the model on delayed functional recovery, respectively. *Results*. Delayed functional recovery was apparent in 76 (39%) people. Higher age, female sex, and higher BMI were all independent risk factors for delayed functional recovery (AUC (95%-CI); 0.72 (0.65–0.80)), whereas blood loss (OR (95%-CI); 1.00 (0.99–1.01)), tourniquet time (OR = 1.00 (0.98–1.02)), and postoperative morphine use (OR = 0.88 (0.37–2.06)) did not statistically improve the predictive value of the model, while surgical experience did (OR = 0.31 (0.16–0.64); AUC = 0.76 (0.69–83)). *Conclusions*. Surgery-related factors contribute little to the patient-related characteristics in a predictive model explaining delayed functional recovery after TKA in daily orthopaedic practice.

## 1. Introduction

Observed inpatient functional recovery is an important outcome measure after total knee arthroplasty (TKA). It is related to length of hospital stay [1], associated with the perioperative experience [2], indicative of functional recovery after hospital discharge [3, 4], and complementary to self-report data [5], predictive of discharge location [6]. But it is, most importantly, highly relevant to the patient. Understanding which factors are associated with inpatient functional recovery after TKA could allow health care providers to anticipate and intervene with more tailored and personalized treatment along with better guidance of patients both during and after the hospital stay.

To date, however, research on predicting inpatient functional recovery is scarce. Researchers generally aim to predict length of hospital stay rather than functional recovery [1, 7], often with little success. The lack of success might be explained by the fact that length of hospital stay is strongly influenced by logistical factors, rather than the actual functional recovery of the patient [8]. Thus, inpatient functional recovery might be more directly influenced by patient characteristics and surgical factors. In this study, we were particularly interested in blood loss, tourniquet time, morphine use, and surgical experience as these have been associated with variations in recovery trajectories after TKA [1, 7, 9–11]. Therefore, the purpose of this study was to evaluate the predictive value of patient characteristics and surgical factors on inpatient functional recovery in a state-of-the-art clinical orthopaedic practice.

To do so, we studied the predictive value of patient characteristics (i.e., age, sex, and BMI) on the inpatient functional recovery of people undergoing elective TKA. Consequently, we evaluated whether the addition of surgical factors (i.e., blood loss, tourniquet time, morphine use, or surgical experience) significantly improved the predictive value of the model.

## 2. Material and Methods

### 2.1. Design and Participants

In this prospective cohort study the functional recovery of 193 consecutive people undergoing TKA was monitored by physiotherapists in the Inpatient Orthopaedic Department of the Porter Adventist Hospital in Denver (CO) from February until June 2012. All 11 physiotherapists participating in this study were first trained on completing the Iowa Levels of Assistance scale (ILAS) and recording scores by use of video cases. The only eligibility criterion for this study was that patients underwent TKA to produce a generalizable and typical study sample. All a priori selected variables were extracted from the medical records. Medical record reviewing was done according to the recommendations of Worster and Haines [12]. Data on perioperative variables were extracted from the computerized medical charts onto standardized computerized data-entry forms by two persons, TH (aware of study goals) and EH (unaware of study goals). Both persons were trained at extracting the data [13]. Unambiguous rules regarding the management of missing/conflicting data were established and the agreement of random samples of the extracted data (*n* = 5) was checked [12]. Data extraction from free-text formats was avoided as much as possible. Discrepancies on entered data were resolved by consensus meetings. In case no consensus was reached, a third person made the final call (JSL). For the data entry, we used the Epidata software (epidata.dk). The Institutional Review Board of the Porter Adventist Hospital approved the study, including the waiver for informed consent (Study ID: 1391).

### 2.2. Surgical Care and Postoperative Rehabilitation

Patients scheduled to undergo TKA were medically cleared by their primary care physician or by a hospital internist. Intraoperatively, tourniquets were routinely used during the procedure and total knee components were cemented in place. Physiotherapy and occupational therapy were initiated immediately postoperatively to address range of motion, gait training, muscle strengthening, and activities of daily living. Postoperative pain was managed using multimodal analgesia including oral narcotic pain medication, nonnarcotic pain medication, and nonsteroidal anti-inflammatory medication (please see Table 1). Intravenous or intramuscular narcotic medication is available for breakthrough pain. The goal was to discharge patients within 3 days after surgery.

### 2.3. Outcome Measures

#### 2.3.1. Dependent Variable

Inpatient functional recovery was monitored by use of the ILAS. The ILAS assesses the amount of assistance that is needed to safely perform four activities of daily life (namely, supine to sit, sit to stand, walking, and stair climbing) [14, 15]. These four activities are considered functional milestones and essential for living independently after surgery. The ILAS was scored daily per transfer on an ordinal scale ranging from 0 (independent, no assistance or supervision necessary to safely perform the activity with or without assistive devices, aids, or modifications) to 6 (test was not attempted due to medical or safety reasons), resulting in a total score range from 0 to 24. With a score of 6 or less, the patient was considered functionally recovered. The interrater reliability of the total ILAS score is good (ICC = 0.98) [14, 15].

#### 2.3.2. Independent Variables

The studied independent variables can be grouped into preoperative, perioperative, and postoperative variables. For the preoperative data, we extracted sociodemographic variables, namely, age, sex, BMI, marital state (single/married), living situation, and work situation (paid job/unemployed/retired). For the perioperative data, we extracted years of surgical experience of the eight surgeons, type of sedation (general, femoral (continuous versus one-shot), and/or spinal), surgical blood loss (in mL; counted according to the amount of blood in suction bags), tourniquet time (in minutes), and complications. For the postoperative data, prescribed pain medication (i.e., morphine use: yes/no) was extracted from the medical chart.

### 2.4. Data Analysis

Descriptive statistics were used to describe the study population, the variables of interest, and the number of missing values. To determine the bivariate relationship between delayed inpatient functional recovery (yes or no) and the independent variables (i.e., surgical experience, blood loss, tourniquet time, and morphine use), we used univariate logistic regression modelling. Surgical experience was dichotomized by use of median split, resulting in a cut-off of 20 years. We coded patients as having delayed inpatient functional recovery if either they did not reach ILAS score of ≤6 at discharge or it took three or more days (including the surgery day) to reach functional independence. Consequently, we built a two-staged logistic regression model to study the corrected relation between inpatient functional recovery and the independent variables [16]. First, we built a multivariable, logistic regression base model that studied the impact of age, sex, and BMI on inpatient functional recovery. Then, we complemented the base model with each of the four independent variables of interest, creating four new models. By use of the Wald tests, we evaluated whether adding blood loss, tourniquet time, morphine use, or surgical experience to the base model significantly (*P* < 0.05) improved the model. Odds ratios were calculated to investigate the independent predictive relevance of the putative factors. Area under the receiver operating curve (AUC) was calculated to determine the predictive value of the models (0.5–0.7 is poor, 0.7–0.9 is good, and >0.9 is excellent).

To test the robustness of our analyses, we performed three sensitivity analyses. First, we recoded the dependent variable as delayed recovery after ≥4 days instead of ≥3 days. Second, we use the ILAS score obtained at day 1 as a proxy for early functional recovery. And third, we used length of hospital stay as the dependent variable in our analyses. For the last two analyses, linear instead of logistic regression techniques were used. Statistical analyses were carried out using Stata/IC 13.

## 3. Results

The studied sample consisted of 193 people with a mean (standard deviation) age of 65 (10) years and BMI of 30 (6) kg/m^2^; the majority (64%) of the population was female. Seventy-six (39%) people reached functional independence after ≥3 days and 32 (17%) people after ≥4 days. A total of 7 patients did not reach functional independence as defined by an ILAS score of ≤6 within 3 days yet were discharged to home (*n* = 3), skilled nursing facility (*n* = 3), or inpatient rehabilitation center (*n* = 1). One complication was documented, namely, myocardial infection. Other characteristics are described in detail in Table 1. No discrepancies were found between the two data extractors. Missing values were negligible; 98% of the cases had less than four missing values. The blood loss variable had the most missing values (*n* = 6/3%). Due to the low number of missing values, complete case analysis was deemed appropriate.

### 3.1. Do Patient Characteristics Predict Inpatient Functional Recovery?

In the base model, we tested the association between patient characteristics and inpatient functional recovery. We found that age (OR: 1.08 (1.04–1.12)), sex (OR: 2.05 (1.05–4.02)), and BMI (OR: 1.13 (1.06–1.20)) are all independently associated with delayed functional recovery. That is, patients who are of higher age, of female sex, and/or with a high BMI are at risk for delayed functional recovery. Area under curve (AUC) (95%-CI) was 0.72 (0.65–0.80).

### 3.2. Do Surgical Factors Predict Inpatient Functional Recovery?

We were unable to demonstrate bivariate relationships between delayed functional recovery and surgical experience (odds ratio (OR) [95% confidence interval]: 0.36 [0.20–0.66]), blood loss (OR: 1.00 (0.99–1.01)), tourniquet time (OR: 0.99 (0.98–1.01)), and postoperative morphine use (OR: 0.95 (0.45–2.01)). Adding blood loss (OR: 1.00 (0.99–1.01)), tourniquet time (OR: 1.00 (0.98–1.02)), or postoperative morphine use (OR: 0.88 (0.37–2.06)) to the model did not statistically significantly improve the predictive value of multivariable logistic regression model (all *P* > 0.05), respectively, AUC = 0.73 (0.65–0.81) (blood loos), AUC = 0.73 (0.65–0.81) (tourniquet time), and AUC = 0.72 (0.65–0.80) (morphine). However, adding the factor orthopaedic experience (>20 years) (OR: 0.31 (0.16–0.64)) to the model did improve the model's fit significantly (*P* < 0.01); AUC = 0.76 (0.69–0.83).

### 3.3. Sensitivity Analyses

Sensitivity analyses yielded similar nonsignificant results for blood loss, tourniquet time, and morphine use in the models explaining delayed functional recovery (≥4 days), the functional recovery at “day 1,” or the length of hospital stay (Table 2).

## 4. Discussion

Basic patient characteristics explain, to some extent, the postoperative inpatient functional recovery after surgery. Adding surgical factors, like surgical experience, blood loss, tourniquet time, and postoperative morphine utilization to the equation, adds little to a preoperative patient-characteristic model predicting inpatient functional recovery in people undergoing TKA surgery in typical, daily orthopaedic practice. Moreover, no relationship between the surgical variables and length of hospital stay could be established.

A strength of this study was the adequate ratio of outcomes to putative predictors for delayed functional recovery (76 : 7). This ratio was greater than 5 (the lowest acceptable limit) [17], indicating that the models were sufficiently powered. Moreover, our preoperative model was well in line with the literature, implying that the included study sample is likely generalizable. There were some limitations to this investigation. First, all data was collected in a single centre, impacting study's generalizability. Yet, considering that none of the studied associations showed any tendency or trend towards statistical significance, it seems unlikely that our data would be opposed in other centres using similar clinical pathways. Another possible limitation was that we found very little variation in the amount of surgical blood loss. Perhaps this was caused by the method used in measuring surgical blood loss, namely, by determining the amount of blood in suction bags. Likely, the true amount of blood loss is higher. Furthermore, we used the ILAS to assess functional recovery. The tool might have two limitations: (1) it may have too many scoring options [18] and (2) it may have difficulty detecting delays in discharge due to medical complications (e.g., wound leakage). On the other hand, we believe the ILAS is currently the best instrument for postoperative recovery after TKA because it measures aspects relevant to regaining base mobility and functional independence that should be key considerations for patient discharge [19]. Finally, inherent to the nature of the study design, prescription of morphine to patients was not randomized and concealed but rather decided upon by the surgeon. Nevertheless, we were not studying the effect of interventions, but we wanted to explain the variance encountered in inpatient functional recovery after TKA. Considering that the utilized surgical and rehabilitation protocol in the centre was straightforward and in line with current recommendations, our prediction model will likely be valid in most modern orthopaedic centres.

Initially we built a preoperative model, comprising age, sex, and BMI, to predict inpatient functional recovery. In line with the available literature, which is scarce, we found that a higher age, being female, and greater BMI were associated with delayed inpatient functional recovery [7]. Our data suggested that adding blood loss, tourniquet time, or morphine use to the preoperative model had no added value to predict differences in patients' functional recovery compared to the preoperative model alone. For tourniquet use, our findings shed new light on a controversial topic in TKA surgery. A recent meta-analysis showed that true blood loss in TKA was not reduced using a tourniquet; however, they also demonstrated that tourniquet use might be associated with more thromboembolic events and wound complications [11]. Another study showed that tourniquet use negatively impacts long term knee range of motion [9]. Nevertheless, early physical activation and mobilization to recover functional activity and abilities are key in the recovery after TKA [8, 20]; and tourniquet time, in the range as seen in this study, did not seem to impact that. For blood loss, we also concluded that, for the range encountered in this study, there was no indication that blood loss was associated with inpatient functional recovery, a finding in line with Husted et al. [1]. Furthermore, for morphine use, we again found no significant relationship with inpatient functional recovery. This may be surprising to some, as such opioids are relatively ineffective for severe movement-associated pain and are associated with significant side effects such as nausea and vomiting [10]. However, morphine prescribed along the clinical algorithms as used in this study did not impact functional mobility in this group of patients. This finding is somewhat in line with those of Holm et al., who demonstrated that postoperative pain levels had only limited impact on functional recovery beyond the first day [21]. Finally, long term experience (>20 years) might be associated with faster inpatient functional recovery, although we were unable to replicate this finding in our sensitivity analyses. Perhaps this finding can be explained by the fact that the more experienced surgeon has more familiarity with surgical techniques and postoperative care. Possibly, a more experienced surgeon engenders more confidence from both patients and hospital staff.

Hippocrates (ca. 460-377 BC) said “*It is more important to know what sort of person has a disease than to know what sort of disease a person has.*” If we replace disease with surgery, this statement seems to fit our data nicely. After all, surgery-related factors appear to contribute little to the patient-related characteristics in our model explaining delayed functional recovery after TKA in daily orthopaedic practice.

## Figures and Tables

**Table 1:** Characteristics of study population (*n* = 193)^*^.

Age	65 (10) years
Sex, %♀	64%
BMI	30 (6) kg/m^2^
Marital status	
Married	71%
Not married	29%
Job status	
Paid job	38%
Retired	57%
Unemployed	6%
Anaesthesia	
Femoral	
Continuous	70%
One-shot	30%
General	7%
Spinal	93%
Blood loss, median (IQR)	100 (100-100) mL
Tourniquet time	49 (15) minutes
Postoperative pain analgesic	
Acetaminophen	35%
Ketorolac	2%
Fentanyl	99%
Morphine	18%
Hydromorphone	79%
Length of stay, median (IQR)	2 (2-3) days
Functional recovery, median (IQR)	2 (2-3) days
Discharge location	
Home	179 (93%)
SNF	12 (6%)
Inpatient rehab	2 (1%)

^∗^Values are mean (standard deviation), unless indicated otherwise. BMI = body mass index, IQR = interquartile range, and SNF = skilled nursing facility.

**Table 2:** Sensitivity analyses on inpatient functional recovery.

	Delayed functional recovery^†^		Functional recovery at day 1^*^		Length of hospital stay	
	OR (95-CI)	AUC (95%-CI)	Coefficient	*R* ^2^	Coefficient	*R* ^2^
Base model						
Age	**1.09** (1.04–1.15)	0.75 (0.65; 0.86)	**0.02** (−0.01–0.05)	0.06	**0.02** (0.01–0.03)	0.10
Sex	0.97 (0.41–2.31)		**0.95** (0.24–1.65)		**0.11** (−0.09–0.30)	
BMI	**1.15** (1.07–1.24)		**0.07** (0.01–0.12)		**0.03** (0.01–0.04)	
Complementary model						
+ Blood loss	1.00 (0.99–1.01)	0.76 (0.65; 0.86)	0.00 (0.00–0.01)	0.07	0.00 (−0.00-0.00)	0.11
+ Tourniquet time	1.00 (0.97–1.03)	0.77 (0.67; 0.87)	−0.00 (−0.03–0.02)	0.06	0.00 (−0.00–0.01)	0.10
+ Morphine use, yes	1.00 (0.31–3.20)	0.75 (0.65; 0.86)	−0.70 (−1.58–0.19)	0.08	0.08 (−0.16–0.33)	0.10
+ Experience, >20 y	**0.31** (0.16–0.64)	0.76 (0.66; 0.86)	−0.54 (−1.26–0.17)	0.08	−0.17 (−0.36–0.27)	0.11

^†^Delayed functional recovery was apparent if ILAS score >6 after ≥4 days, ^*^ILAS score after day 1. BMI = body mass index, OR = odds ratio, and AUC = area under the curve.
